# Molecular Mechanisms of Heatstroke: Pathophysiology and Cell Death Pathways

**DOI:** 10.14789/ejmj.JMJ25-0023-P

**Published:** 2025-10-22

**Authors:** RICARD FERRER, TOSHIAKI IBA

**Affiliations:** 1 Intensive Care Department, Hospital Universitari Vall d’Hebron Universitat Autònoma de Barcelona, Barcelona, Spain; 1 Intensive Care Department, Hospital Universitari Vall d’Hebron Universitat Autònoma de Barcelona, Barcelona, Spain; 2 Faculty of Medical Science, Juntendo University, Chiba, Japan; 2 Faculty of Medical Science, Juntendo University, Chiba, Japan

**Keywords:** heat stroke, damage-associated molecular patterns, cell death, mitochondria, leukocyte

## Abstract

Heatstroke is a severe, life-threatening condition characterized by a core body temperature exceeding 40°C, accompanied by central nervous system dysfunction such as confusion, seizures, or coma. It often presents with systemic inflammation and multi-organ failure, including injury to the liver, kidneys, brain, and coagulation system. The molecular pathogenesis of heatstroke is multifactorial, involving a cascade of intracellular and extracellular events triggered by excessive heat. These include protein denaturation, loss of cellular homeostasis, oxidative stress, mitochondrial dysfunction, and the release of damage-associated molecular patterns (DAMPs) that activate immune responses. In parallel, regulated cell death pathways such as apoptosis, necroptosis, pyroptosis, and ferroptosis are initiated, amplifying tissue injury. Key molecular mediators include heat shock proteins, proinflammatory cytokines, and reactive oxygen species. This review summarizes current understanding of the molecular and cellular mechanisms underlying heatstroke, highlighting potential biomarkers and therapeutic targets that may improve diagnosis, prognostication, and clinical management of this increasingly relevant condition.

## Introduction

Spain faced an exceptionally intense and early heatwave during the summer of 2025, resulting in unprecedented temperatures and a marked rise in heat-related fatalities. Heatstroke is the most severe form of heat-related illness and is increasingly recognized as a serious global public health issue, especially in the context of rising environmental temperatures and more frequent extreme weather events. While it begins with a failure of the body’s thermoregulatory mechanisms, heatstroke rapidly progresses to cause widespread cellular and molecular disturbances. These disturbances include protein misfolding, oxidative stress, inflammatory responses, and the activation of various forms of programmed cell death, ultimately leading to tissue damage and multi-organ failure^1, 2)^. In this review, we introduce the current understanding of the molecular and cellular mechanisms of heatstroke.

## Protein denaturation and heat shock response

One of the earliest cellular consequences of extreme heat exposure is protein denaturation and aggregation. Because proteins are highly sensitive to temperature, even mild hyperthermia can disrupt their three-dimensional structure, impairing function and promoting toxic aggregation^3)^. In response, cells activate the heat shock response (HSR), a conserved defense mechanism regulated primarily by heat shock transcription factor 1 (HSF1). Upon activation, HSF1 translocates to the nucleus and induces the expression of heat shock proteins (HSPs), especially HSP70^4, 5)^. These molecular chaperones assist in refolding denatured proteins, prevent further aggregation, stabilize the cytoskeleton, and protect membrane integrity. However, in cases of severe or prolonged heat stress, the HSR may become overwhelmed, allowing misfolded proteins to accumulate and initiate downstream cellular damage pathways^6)^.

## Oxidative stress and mitochondrial dysfunction

Hyperthermia triggers profound oxidative stress, a central mechanism in the pathogenesis of heatstroke. Excessive heat leads to the overproduction of reactive oxygen species (ROS), highly reactive molecules derived from oxygen, primarily generated within the mitochondria during cellular respiration. Under normal conditions, ROS are tightly regulated and serve physiological roles in signaling. However, during heat stress, this regulatory balance is lost, resulting in ROS accumulation that damages essential cellular components, including lipids, proteins, and nucleic acids^7, 8)^.

A hallmark of heat-induced oxidative injury is lipid peroxidation, wherein ROS attack polyunsaturated fatty acids in cell membranes. This process disrupts membrane fluidity and integrity, increasing permeability and leading to cell swelling, ion imbalance, and eventual lysis. To counteract oxidative damage, cells rely on endogenous antioxidant systems, including glutathione (GSH) and glutathione peroxidase 4 (GPX4), which reduce lipid hydroperoxides and detoxify ROS. However, under sustained heat stress, these defense systems become depleted or dysfunctional, tipping the balance toward uncontrolled oxidative damage and triggering regulated forms of cell death, such as ferroptosis^9)^.

Additionally, oxidative stress contributes directly to mitochondrial dysfunction, which amplifies cellular injury. Damaged mitochondria leak cytochrome c, a pro-apoptotic protein that activates the intrinsic (mitochondrial) pathway of apoptosis. Simultaneously, mitochondrial damage further promotes ROS production, creating a self-perpetuating cycle of oxidative injury, inflammation, and cell death^10)^. This vicious loop plays a key role in the progression of multi-organ dysfunction in severe heatstroke.

## Inflammatory response and endotoxemia

Heat exposure disrupts the intestinal epithelial barrier, allowing translocation of bacterial endotoxins such as lipopolysaccharides (LPS) into the circulation^11)^. This stimulates a systemic inflammatory response, characterized by elevated levels of proinflammatory cytokines such as interleukin-6 (IL-6), tumor necrosis factor-alpha (TNF-α), and interleukin-1β (IL-1β)^12)^. This cytokine storm resembles sepsis, leading to hypotension, capillary leak, and multi-organ dysfunction. Nuclear factor-*kappa* B (NF-κB), a central transcription factor, regulates many of these inflammatory mediators and contributes to the progression of organ injury^13)^. Systemic inflammation promotes coagulopathy, including disseminated intravascular coagulation (DIC), by activating endothelial cells, platelets, and clotting factors^14)^.

## Cell death mechanisms in heatstroke

**Figure 1:**
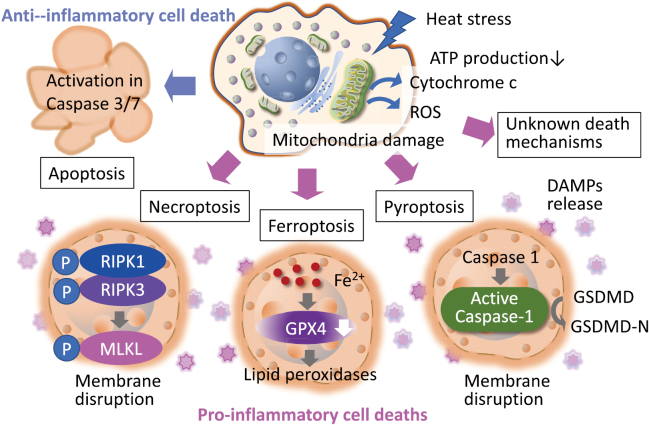
Cell death mechanisms in heatstroke The figure illustrates the major programmed cell death pathways activated under heat stress and their molecular features. Heat-induced mitochondrial damage leads to increased reactive oxygen species (ROS), ATP (adenosine triphosphate) depletion, and cytochrome c release, triggering apoptosis via caspase-3/7 activation, which is generally non-inflammatory. In contrast, ferroptosis, necroptosis, and pyroptosis are pro-inflammatory cell deaths associated with membrane disruption and the release of damage-associated molecular patterns (DAMPs). Ferroptosis is characterized by iron (Fe^2+^) accumulation, glutathione peroxidase 4 (GPX4) suppression, and lipid peroxidation. Pyroptosis involves Gasdermin D (DSDMD) cleavage, while necroptosis proceeds via membrane rupture. Together, these pathways amplify inflammation and tissue injury in heatstroke. RIPK: receptor-interacting protein kinase, MLKL: Mixed lineage kinase domain-like protein

Several forms of regulated cell death contribute to the tissue injury and organ dysfunction observed in heatstroke. Apoptosis, a caspase-dependent and typically non-inflammatory process, is triggered by oxidative stress via the mitochondrial pathway, leading to the activation of Bax (Bcl-2-associated X protein) /Bak (Bcl2 antagonist/killer), release of cytochrome c, and subsequent caspase-3 activation^10, 15)^. In contrast, necroptosis is a caspase-independent but pro-inflammatory cell death mechanism mediated by receptor-interacting protein kinases (RIPK [receptor-interacting protein kinase] 1 and RIPK3) and mixed lineage kinase domain-like protein (MLKL), resulting in cell membrane rupture and the release of damage-associated molecular patterns (DAMPs) that intensify the inflammatory response^16)^. Ferroptosis, another distinct form of regulated cell death, is characterized by iron-dependent lipid peroxidation and mitochondrial shrinkage, and is driven by the accumulation of ROS and downregulation of GPX4 (glutathione peroxidase 4), particularly contributing to hepatic and neuronal injury in heatstroke^9, 17)^. Additionally, pyroptosis, a caspase-1 or -11-dependent process involving gasdermin-mediated pore formation, causes inflammatory cell lysis and the release of cytokines like IL-1β. Emerging research has also identified PANoptosis (a prominent innate immune, inflammatory, and lytic cell death), a highly integrated mechanism that combines features of pyroptosis, apoptosis, and necroptosis, underscoring the synergistic nature of cell death pathways during severe systemic inflammation such as that seen in heatstroke^18, 19)^ (Figure 1). These interconnected pathways collectively drive inflammation, disrupt tissue integrity, and contribute to multiorgan failure in severe cases.

## Impaired thermotolerance and susceptibility factors

Several intrinsic factors impair thermotolerance, making individuals more susceptible to heatstroke. For example, aging reduces HSP induction and antioxidant reserves. Dehydration exacerbates oxidative stress and intestinal permeability^20, 21)^. Transcription factors such as nuclear factor erythroid 2 (NFE2), which regulates antioxidant gene expression, and Poly-ADP-ribose polymerase 1 (PARP1), which is involved in DNA repair, are critical in modulating cellular survival during heat stress^22)^.

## Systemic effects and multi-organ dysfunction

Prolonged heat exposure and unresolved cellular injury in heatstroke lead to systemic inflammation and multi-organ dysfunction. The central nervous system is often the first to be affected, with oxidative stress and neuronal apoptosis resulting in cerebral edema, seizures, and coma^23)^. The liver sustains mitochondrial and ferroptotic damage, leading to transaminase elevations, hyperbilirubinemia, and coagulopathy, which can progress to acute liver failure^24)^. Renal dysfunction is common, driven by rhabdomyolysis, hypovolemia, and oxidative injury, resulting in acute tubular necrosis and potentially requiring dialysis^25)^. The heart may suffer from arrhythmias, myocardial injury, and circulatory instability due to endothelial injury and systemic inflammation^26)^.

A unifying feature of heatstroke-related organ failure is endothelial barrier disruption, which promotes vascular leakage, tissue edema, and microthrombi formation. These changes impair tissue perfusion and oxygen delivery. Alongside immune overactivation and the release of DAMPs, this state can mimic or trigger DIC, contributing to irreversible shock if not rapidly addressed^14)^. Thus, the multi-organ injury in heatstroke reflects a complex interplay of oxidative stress, inflammation, coagulation disturbances, and cellular death pathways. Prompt recognition and intervention are critical to prevent fatal outcomes.

## Conclusion

Heatstroke is a multifactorial condition characterized not only by elevated body temperature but also by systemic inflammation, organ dysfunction, and cellular injury. It is a multifaceted disorder driven by protein denaturation, oxidative stress, systemic inflammation, and multiple programmed cell death pathways. The integration of heat shock responses, mitochondrial damage, and immune dysregulation culminates in catastrophic organ failure. Understanding the molecular mechanisms provides a foundation for identifying new diagnostic biomarkers and therapeutic targets to improve outcomes in this increasingly prevalent condition.

## Author contributions

RF and TI wrote and reviewed the manuscript. Both authors read and approved the final manuscript.

## Conflicts of interest statement

The authors declare that they have no conflict of interest. T. Iba that one of the JMJ Editorial Board members was not involved in the peer review or decision-making process for this paper.

## References

[1] Bouchama A, Knochel JP: Heat stroke. N Engl J Med, 2002; 346: 1978-1988. 12075060 10.1056/NEJMra011089

[2] Leon LR, Helwig BG: Heat stroke: role of the systemic inflammatory response. J Appl Physiol (1985), 2010; 109: 1980-1988. 10.1152/japplphysiol.00301.2010 20522730

[3] Baler R, Zou J, Voellmy R: Evidence for a role of Hsp70 in the regulation of the heat shock response in mammalian cells. Cell Stress Chaperones, 1996; 1: 33-39. 10.1379/1466-1268(1996)001<0033:efaroh>2.3.co;2 PMC313015 9222587

[4] Hu C, Yang J, Qi Z, Wu H, et al: Heat shock proteins: Biological functions, pathological roles, and therapeutic opportunities. MedComm (2020), 2022; 3: e161. 35928554 10.1002/mco2.161 PMC9345296

[5] Liu Z, Chen J, Hu L, et al: Expression profiles of genes associated with inflammatory responses and oxidative stress in lung after heat stroke. Biosci Rep, 2020; 40: BSR20192048. 32436952 10.1042/BSR20192048 PMC7276522

[6] Stetler RA, Gan Y, Zhang W, et al: Heat shock proteins: cellular and molecular mechanisms in the central nervous system. Prog Neurobiol, 2010; 92: 184-211. 20685377 10.1016/j.pneurobio.2010.05.002 PMC2939168

[7] Nakahira K, Kyung SY, Rogers AJ, et al: Circulating mitochondrial DNA in patients in the ICU as a marker of mortality: derivation and validation. PLoS Med, 2013; 10: e1001577. 24391478 10.1371/journal.pmed.1001577 PMC3876981

[8] Marchi S, Guilbaud E, Tait SWG, Yamazaki T, Galluzzi L: Mitochondrial control of inflammation. Nat Rev Immunol, 2023; 23: 159-173. 35879417 10.1038/s41577-022-00760-x PMC9310369

[9] Ji J, Gao J, Wang C, Ouyang L, Liu Z, Liu Z: Characteristics and Outcome of Exertional Heatstroke Patients Complicated by Acute Hepatic Injury: A Cohort Study. J Clin Transl Hepatol, 2021; 9: 655-660. 34722180 10.14218/JCTH.2021.00084 PMC8516842

[10] Geng Y, Ma Q, Liu YN, et al: Heatstroke induces liver injury via IL-1β and HMGB1-induced pyroptosis. J Hepatol, 2015; 63: 622-633. 25931416 10.1016/j.jhep.2015.04.010

[11] Sun M, Li Q, Zou Z, Liu J, Gu Z, Li L: The mechanisms behind heatstroke-induced intestinal damage. Cell Death Discov, 2024; 10: 455. 39468029 10.1038/s41420-024-02210-0 PMC11519599

[12] Bouchama A, Ollivier V, Roberts G, et al: Experimental heatstroke in baboon: analysis of the systemic inflammatory response. Shock, 2005; 24: 332-335. 16205317 10.1097/01.shk.0000180620.44435.9c

[13] Iba T, Connors JM, Levi M, Levy JH: Heatstroke-induced coagulopathy: Biomarkers, mechanistic insights, and patient management. EClinicalMedicine, 2022; 44: 101276. 35128366 10.1016/j.eclinm.2022.101276 PMC8792067

[14] Wu J, Cheng Z, Yang S, et al: Specific immune landscape of heatstroke distinguished from sepsis and aseptic inflammation. Int J Med Sci, 2025; 22: 1450-1464. 40084252 10.7150/ijms.108212 PMC11898847

[15] Leon LR, Bouchama A: Heat stroke. Compr Physiol, 2015; 5: 611-647. 25880507 10.1002/cphy.c140017

[16] Yu C, Huang Y, Xie J, et al: HMGB1 released from pyroptotic vascular endothelial cells promotes immune disorders in exertional heatstroke. Int J Hyperthermia, 2024; 41: 2378867. 39117343 10.1080/02656736.2024.2378867

[17] Iba T, Helms J, Maier CL, Ferrer R, Levy JH: Mitochondrial dysfunction is a major cause of thromboinflammation and inflammatory cell death in critical illnesses. Inflamm Res, 2025; 74: 17. 39806233 10.1007/s00011-025-01994-w

[18] Hirose T, Hamaguchi S, Matsumoto N, et al: Presence of neutrophil extracellular traps and citrullinated histone H3 in the bloodstream of critically ill patients. PLoS One, 2014; 9: e111755. 25392950 10.1371/journal.pone.0111755 PMC4230949

[19] Goto H, Kinoshita M, Oshima N: Heatstroke-induced acute kidney injury and the innate immune system. Front Med (Lausanne), 2023; 10: 1250457. 37614951 10.3389/fmed.2023.1250457 PMC10442538

[20] Asmara IGY: Diagnosis and Management of Heatstroke. Acta Med Indones, 2020; 52: 90-97. 32291378

[21] Epstein Y, Yanovich R: Heatstroke. N Engl J Med, 2019; 380: 2449-2459. 31216400 10.1056/NEJMra1810762

[22] Zhang Z, Wu X, Zou Z, et al: Heat stroke: Pathogenesis, diagnosis, and current treatment. Ageing Res Rev, 2024; 100: 102409. 38986844 10.1016/j.arr.2024.102409

[23] Powers JH, Scheld WM: Fever in neurologic diseases. Infect Dis Clin North Am, 1996; 10: 45-66. 8698994 10.1016/s0891-5520(05)70285-3

[24] Matsumoto H, Takeba J, Umakoshi K, et al: Successful treatment for disseminated intravascular coagulation (DIC) corresponding to phenotype changes in a heat stroke patient. J Intensive Care, 2019; 7: 2. 10.1186/s40560-019-0359-3 PMC6332900 30675362

[25] Kondo K, Hashiguchi N, Watanabe S, Nishio H, Takazawa Y, Iba T: Mechanism of Acute Kidney Injury in Mild to Moderate Heat-related Illness. Juntendo Med J, 2024; 70: 420-428. 10.14789/ejmj.JMJ24-0013-OA PMC11745825 39840004

[26] Périard JD, Travers GJS, Racinais S, Sawka MN: Cardiovascular adaptations supporting human exercise-heat acclimation. Auton Neurosci, 2016; 196: 52-62. 26905458 10.1016/j.autneu.2016.02.002

